# A Narrative Review of Adhesive Capsulitis with Diabetes

**DOI:** 10.3390/jcm13195696

**Published:** 2024-09-25

**Authors:** Mu-Her Chen, Wen-Shiang Chen

**Affiliations:** 1 Department of Medical Education, National Taiwan University Hospital, Taipei 100229, Taiwan; 112ntuh@gmail.com; 2 Department of Physical Medicine and Rehabilitation, National Taiwan University Hospital, Taipei 100229, Taiwan

**Keywords:** adhesive capsulitis, frozen shoulder, diabetes, diabetes mellitus

## Abstract

**Background/Objectives:** To update the perspectives of the association between diabetes (DM) and adhesive capsulitis (AC). **Methods:** Our findings were summarized in a narrative review. We searched PubMed, Embase, and Consensus databases, using keywords such as “diabetes”, “adhesive capsulitis”, and “frozen shoulder”, for articles published from January 2015 to June 2024, covering both type 1 and type 2 DM. **Results:** After compiling relevant articles on DM-related AC published since 2015, we found that most studies show an increased prevalence of AC in DM patients, ranging from 3 to 10 times. A longer duration of DM is a risk factor for AC. Whether T1DM or prolonged insulin use will increase the risk of AC is still controversial. Poor blood sugar control seems to increase the risk of AC. Recent studies also show a correlation between blood sugar levels and the prevalence of AC. Cytokines, such as IL-6, IL-8, TNF-α, VEGF, and AGEs, related to inflammation and fibrosis may contribute to the pathophysiological processes of AC. **Conclusions:** Recent research findings have revealed new perspectives divergent from past notions, while also presenting some topics worthy of exploration. Due the close relationship between DM and AC, clinicians need to be alert to the presence of AC, especially early stage, in DM cases, and control the blood glucose level to reduce the risk of AC. Further research is still needed to provide better prevention and management for DM patients with AC.

## 1. Introduction

ISAKOS Upper Limb Committee classified adhesive capsulitis (AC), also known as frozen shoulder (FS), into primary idiopathic and secondary AC [1]. Primary AC occurs without any identifiable trauma or direct causes, though the patient may have conditions like diabetes mellitus (DM) or thyroid disorders that are associated with stiffness. On the other hand, secondary AC refers to joint stiffness resulting from a known underlying cause, such as trauma, infection, or inflammatory disorders.

Traditionally, AC is described in ‘three clinico-pathological stages’, which can be divided as follows: the freezing stage, the frozen stage, and the thawing stage, each characterized by distinct symptoms of pain, pain and stiffness, and stiffness, respectively [2]. While AC is often self-limiting around 1 to 3 years [3], diagnosis in the early freezing stage could be challenging. The diagnosis of AC is usually based on clinical evaluation, including pain and stiffness, especially the passive range of motion limitation in different directions. Although imaging is not essential for diagnosing AC, it still helps clinical physicians to rule out other diagnoses. The most common imaging tools for AC diagnosis are X-ray, MRI, and ultrasound (US). X-ray is a basic tool used to rule out bony conditions such as osteoarthritis or fracture of the shoulder joint. Thickening of the glenohumeral joint capsule in the axillary pouch (AP), thickening of the coracohumeral ligament (CHL), obliteration of the subcoracoid fat triangle, and synovitis of the rotator interval are typical findings for MRI. Capsular thickening of more than 4 mm in the axillary recess is considered a highly sensitive diagnostic criterion for MRI [4]. Thickening of the CHL and AP, as well as hypoechoic material at the rotator interval and increased Doppler vascular flow in the rotator interval, are also frequently found by ultrasound [4,5]. However, these imaging findings and clinical symptoms may not be obvious in the early stage, which relies heavily on the experience of clinical physicians. Therefore, establishing the possible risk factors of AC could help the early diagnosis and treatment.

AC is an inflammatory and fibrosing condition of the shoulder, causing gradual pain and limited movement in the joint. Although the pathology and etiology of AC remains unclear, a few related risk factors have been reported. In addition to common factors like female gender, obesity, DM, and thyroid disorders [6], some studies have indicated associated conditions such as gout [7], osteoarthritis [8], high blood lipids [9], HLA-B27 [10], and psychiatric disorders [11].

Taking DM as an example, past studies have estimated that the prevalence of AC ranges from 10.8 to 30% [12], significantly higher than that of the general population. Additionally, symptoms of AC in DM patients are often more severe, requiring prolonged conservative treatment, and may require surgical treatment more often than non-diabetic patients [13,14]. Recent studies suggest that conservative treatments, such as corticosteroid injections, can serve as the first-line therapy for diabetic AC patients, although the outcomes are generally less favorable compared to those of non-diabetic patients [15]. Surgical interventions like arthroscopic capsular release (ACR) have been proven effective in improving shoulder range of motion and alleviating pain symptoms in diabetic AC patients [16]. While some recent studies indicate that the prognosis following ACR remains worse for diabetic patients, particularly in terms of internal rotation and forward flexion, ACR is still widely recognized as the most effective invasive treatment [17].

Recent studies continue to support the previous conclusion for the treatment of diabetic AC. However, any treatment for diabetic AC must be accompanied by proper blood sugar control [14]. Additionally, studies on the prevalence of AC- and DM-associated risk factors have shown significant discrepancies in recent years. Thus, more a detailed investigation and a discussion on the correlation between AC prevalence and DM are warranted. Our narrative review integrates current multifactorial observational and experimental studies from selective literature, providing a comparison of the impact of type 1 DM, type 2 DM, glycated hemoglobin (HbA1c), and complications of DM on the prevalence of AC, as well as updates on new perspectives and trends from various studies over the past decade.

## 2. Materials and Methods

We conducted database searches using keywords such as “diabetes”, “adhesive capsulitis”, and “frozen shoulder”. The databases consulted included PubMed, Embase, and Consensus. We restricted our searches to English language articles published between January 2015 and September 2024, without limiting the type of article. After excluding articles with incomplete content or those not indexed in PubMed, the results from these searches included a total of 38 articles on the association between DM and the prevalence of AC. Discussions on type 1 DM (T1DM), blood glucose levels, DM duration, and pathophysiology are presented as a narrative review.

## 3. Results

### 3.1. Prevalence of AC in DM Patients

There are several studies providing evidence on the association between DM and AC, including two cross-sectional studies [18,19], three retrospective studies [20,21,22], and two prospective study [23,24] (Table 1).

It is generally believed that DM is one of the most important risk factors for AC. The overall prevalence of AC is 2–5% [25,26], with most patients being aged between 40 and 60 years at diagnosis [27]. Zreik conducted a meta-analysis, integrating thirteen studies published before 2014, showing that the prevalence of AC among DM patients was approximately 13.4%, indicating a risk approximately five times higher than that of non-diabetic individuals [12].

Most studies published since 2015 still largely support this viewpoint. A systematic review conducted by Dyer reaffirmed previous perspectives and demonstrated that DM increases the risk of AC by 3.69 times compared to non-diabetic patients [28]. Another study by Inayat reported an AC prevalence of approximately 41.3% among DM patients [29]. Additionally, a study by Kim investigated 3.47 million individuals in a large health insurance database, and the results also supported the idea that DM increases the risk of developing AC [20]. However, Jacob reported no statistically significant relationships between DM and AC [22]. He indicated that the risk of AC is more pronounced in individuals with severe DM, therefore, the difference in statistical results may arise from the small sample size and mild DM cases. The severe DM mentioned above could be explained as a high blood sugar level or long-term DM, and this issue will be discussed below.

**Table 1:** Summary of studies about the prevalence of AC in DM patients and the prevalence of DM in AC patients.

Study	DM Types	Methods	Results
Inayat et al. (2017) [29]	Not distinguished	A cross-sectional study on 80 patients with diabetes.	(1) Insulin-dependent patients: 1.93 times more likely to have AC (1.96 after adjusting for HbA1c). (2) Patients on oral hypoglycemic drugs: 1.5 times more likely to develop AC than those not on insulin or oral agents. (3) Insulin users (with or without oral agents): 1.2 times higher rate of AC (4) Poor glycemic control (past 3 months): 1.5 times higher rate of AC. (5) A total of 41.3% of DM patients had AC.
Safran et al. (2017) [19]	Not distinguished	A cross-sectional study on 50 patients with AC.	(1) A total of 8% of AC patients were prediabetic. (2) No patients with AC were found to have DM.
Gundtoft et al. (2018) [21]	Not distinguished	A retrospective study on 34 patients with and 201 patients without diabetes.	(1) Prevalence of AC in DM patients vs. control group: no significant difference (0% vs. 2.6%).
Alhashimi et al. (2018) [23]	Not distinguished	A prospective study on 216 patients with DM.	(1) A total of 90.3% of AC patients had DM. (2) FS often present in chronic DM: 32.3% (1–5 years) and 33.8% (5–10 years). (3) A total of 10–30% of DM patients develop AC, and often less responsive to treatment.
Rai et al. (2019) [24]	Not distinguished	A prospective study on 135 patients with AC.	(1) A total of 15.5% of AC patients were prediabetic. (2) A total of 27.4% of AC patients had DM.
Kim et al. (2023) [20]	T2DM	A retrospective study on 3,471,745 subjects with type 2 DM in NHIS medical checkup.	(1) IR of AC: normal group, 9.453; prediabetes group, 11.912; new-T2DM group, 14.933; T2DM group, 24.3761. (2) Adjusted IR of AC: normal group, 1; prediabetes group, 1.084; new-T2DM group, 1.312; T2DM group, 1.473. * newly diagnosed T2DM = new-T2DM * T2DM (claim history for antidiabetic medication)
Jacob et al. (2023) [22]	Not distinguished	A retrospective study on 8439 patients with and 42,195 patients without AC.	(1) A total of 36.5% of AC patients had DM; 33.9% of normal patients had DM. (2) No significant relationship between DM and AC.
Pandey et al. (2024) [30]	Not distinguished	A prospective study on 158 patients with primary AC.	(1) A total of 37.3% of AC patients were prediabetic. (2) A total of 46.2% of AC patients had DM.

### 3.2. Prevalence of DM in AC Patients

Due to the high correlation and prevalence rates of both AC and DM, discussing the two diseases often gives rise to another interesting question: What is the prevalence of DM among AC patients? If the prevalence was high, it could aid in predicting the diagnosis of DM. Unfortunately, relevant research is scarce. Zreik reviewed five studies conducted before 2014, revealing that the prevalence of DM among AC patients ranged from 20% to 40%, with a mean prevalence of 30% [12]. Although these studies are dated and have limited sample sizes, if this conclusion is true, it could be used for the diabetic screening of AC patients.

In recent years, there have been divergent research findings on this topic (Table 1). Safran and El-Haj found that, out of 50 AC patients, only 4 were diagnosed as prediabetic, with none being diagnosed with DM [19]. A study by Gundtoft also reported the same result [21]. However, Rai showed that, out of 135 AC patients, 21 were diagnosed as prediabetic and 37 were diagnosed with DM [24]. On the other hand, Pandey revealed that, among 158 AC patients, 59 were diagnosed with prediabetes and 73 with DM [30]. In these studies, there was a varying prevalence of prediabetes, ranging from 8% to 15.5% to 37.3%, and the prevalence of DM ranged from 0% to 27.4% to 46.2%. Such significant differences in the statistical figures and conflicting conclusions make this issue more contentious.

Additionally, Alhashimi revealed that AC onset was predominantly observed during the chronic phase of DM, with rates of 32.3% and 33.8% for durations of 1–5 years and 5–10 years, respectively [23]. This result is consistent with the findings of Yian and Juel, indicating that a longer duration of DM is a risk factor for AC [22,31]. Therefore, screening for AC may help to identify potential chronic DM patients, but whether it contributes to early DM diagnosis remains controversial.

### 3.3. Prevalence of AC in T1DM Patients

Research on the association between type 1 DM (T1DM) and AC is relatively scarce. Studies published before 2000 mostly concurred that T1DM increases the prevalence of AC. For example, Arkkila indicated that the prevalence of AC among type 1 and type 2 diabetes mellitus (T2DM) patients was 10.3% and 22.4%, respectively [32]. On the other hand, Bunker (1997) analyzed data from three thousand individuals in Pakistan, reporting that the prevalence of AC in DM patients ranged from 10% to 20%, while the prevalence in T1DM patients was 36% [33]. However, a meta-analysis conducted by Zreik, compiling studies published before 2014, indicated that there was no difference in AC prevalence between T1DM and T2DM patients.

T1DM-related studies published since 2015 have once again overturned the previous viewpoint, including two cross-sectional studies [31,34], one retrospective study [35], and one cross-sectional study [29], focusing on insulin use (Table 2). 

Two studies conducted by Juel and Doria, respectively, reported AC prevalence rates of 59% and 35.1% among T1DM patients [31,34]. The findings significantly exceeded the AC prevalence among general DM patients. Additionally, Yang published a retrospective study involving 160,000 individuals from a China health insurance database [35], the results of which also confirmed the higher AC prevalence among T1DM patients compared to general DM patients. Finally, Inayat indicated that insulin use increases the risk of AC by 1.2 times, contradicting the findings of the meta-analysis by Zreik in 2016 [29].

Finally, a genome-wide association study by Green provided evidence that T1DM is a causal factor for the condition, though it has a weaker association with T2DM [36]. The difference in AC risk can be explained by the earlier age of diagnosis in T1DM compared to the later age of diagnosis in T2DM. Based on these findings, it is reasonable to infer that the risk of AC increases in all DM patients, particularly those with a longer disease duration (such as T1DM patients) and poor glycemic control. Notably, the long-term impact of elevated blood sugar levels on AC risk can span from several years to decades. Therefore, when diagnosing a DM patient with AC, it is crucial to carefully assess their chronic DM history and long-term glycemic control.

Over the past 30 years, research on the association between T1DM and AC has been scarce. Although a meta-analysis published in 2016 consolidated the relevant studies before [12], the current research outlines conflicting viewpoints, suggesting that T1DM is the greatest risk factor for the development of AC. Considering the significant discrepancies in statistical data, with inconsistent conclusions, further relevant studies are needed to continue exploring this topic.

### 3.4. Correlation between AC Prevalence and Insulin Use

Research on the impact of insulin and oral hypoglycemic agents on the risk of AC is relatively limited. A retrospective analysis with statistical review by Yian (2012), which examined 210,000 diabetic patients in Southern California, found that patients using insulin had a 1.93 times higher risk of AC compared to non-insulin-dependent patients [37]. The patients who used only insulin had a 2.8 times higher risk of AC compared to those who did not use either insulin or oral hypoglycemic agents. The patients using oral hypoglycemic agents had a 1.5 times higher risk of AC compared to those who did not use any blood sugar medications.

**Table 2:** Summary of studies about the prevalence of AC in T1DM patients, insulin, and oral hypoglyce-mic agents (OHAs) user.

Study	Methods	Results
Juel et al. (2017) [31]	A cross-sectional study on 105 subjects with T1DM and 73 DM-free subjects.	(1) Point Prevalence of AC in T1DM Patients: 59% (2) Lifetime Prevalence of AC: T1DM group: 76%, diabetes-free group: 14%
Doria et al. (2017) [34]	A cross-sectional study on 943 patients with T1DM.	(1) Prevalence of AC in T1DM: 35.1% (2) Association with Duration: Longer T1DM duration linked to higher AC prevalence. (3) HbA1c Levels: No significant difference between patients with and without AC. (4) Glycemic Control: AC was more frequent in patients with poor control (HbA1c > 8%).
Inayat et al. (2017) [29]	A cross-sectional study on 80 patients.	(1) Prevalence of AC: 41.3% in DM patients. (2) Uncontrolled DM: 1.5 times higher AC rate. (3) Insulin Use: 1.2 times higher AC rate.
Eckert et al. (2021) [18]	A retrospective study on 44,613 subjects with T1DM and 401,816 with T2DM from DPV database.	(1) Prevalence of AC: Higher in T1DM (0.22%) compared to T2DM (0.06%). (2) Blood Sugar Level: Higher blood sugar levels in T1DM were associated with increased AC prevalence.
Yang et al. (2023) [35]	A retrospective study on T1DM (5928 cases and 183,185 controls) and T2DM (48,286 cases and 250,671 controls) from IEU GWAS database.	(1) OR of AC in T1DM: 1.103 (inverse variance weighted), 1.109 (weighted median). (2) T2DM showed no significant association with AC.
Lin et al. (2024) [38]	A retrospective study on 30,412 T2DM patients with metformin and 121,648 T2DM patients without metformin.	(1) T2DM patients on metformin had a significantly higher risk of developing AC (crude HR = 1.205).

In recent years, Inayat, through a cross-sectional study of approximately 3000 individuals in Pakistan, indicated that insulin use increases the risk of AC by 1.2 times [29]. Additionally, a retrospective cohort study by Lin, which analyzed the health insurance database of 121,648 individuals, indicated that patients taking metformin had a significantly higher risk of developing AC compared to those who did not [38]. It is worth noting that many past studies did not evaluate the risk specifically associated with the use of insulin, but instead focused on insulin-dependent diabetes mellitus (Table 2). Nevertheless, the influence of insulin and oral hypoglycemic agents on the prevalence of AC continues to show a significant positive correlation.

### 3.5. Correlation between AC Prevalence and Blood Sugar Levels

We have compiled research from the past decade on the correlation between blood sugar levels and the prevalence of AC. There are three retrospective studies and two cross-sectional studies included here (Table 3).

We have long understood the close association between DM and AC, but whether increased blood sugar levels and uncontrolled DM raise the risk of developing AC has been less explored. A large-scale review conducted by Yian, involving approximately 200,000 individuals, suggested that there is no correlation between blood sugar levels and the prevalence of AC [37]. However, current research is starting to challenge this viewpoint.

Another large-scale review conducted by Chan, involving 24,417 individuals in the United States, found a correlation between blood sugar and prevalence, where each unit increase in HbA1c > 7 raised the prevalence by 2.77% [39]. Two other studies, involving 604 and 80 diabetic patients, respectively, also supported this notion, suggesting that uncontrolled blood sugar increases the risk by 1.5 times [29,40]. Interestingly, Park conducted a study on a population with normal fasting glucose levels and found that AC is positively associated with fasting glucose levels of 90–99 mg/dL, which are currently considered normoglycemic [40].

Additionally, two studies about HbA1c and AC prevalence in T1DM patients have shown conflicting results. Firstly, a cross-sectional study by Doria suggested a 35.1% increase in AC prevalence [34], which showed no positive correlation with HbA1c levels. However, the other study by Eckert, involving approximately forty thousand patients, proposed that higher blood sugar levels corresponded to a higher prevalence rate. He also suggested that glycemic control has a greater impact on AC in T1DM than in T2DM [18].

In summary, most research published after 2015 suggests a positive correlation between blood sugar levels and the prevalence of AC, challenging the previous beliefs and guiding future research for AC prevention.

## 4. Discussion

### 4.1. Pathological Mechanism of AC by DM

Currently, the mechanism of AC remains unclear. However, AC is known to be associated with proliferative, fibroblastic, and inflammatory processes by histological sections, blood data, arthroscopy, and clinical symptoms [24,41]. Histological sections showed an increased number of immune cells, amount of cytokines such as interleukins (IL-6 and IL-8), tumor necrosis factor-alpha (TNF-α) [42], stronger immunostaining to vascular endothelial growth factor (VEGF) [43], and advanced glycation end products (AGEs) [44,45]. These results can reasonably explain the AC findings under arthroscopy that show synovial hyperplasia with vascularization and the subsequent rotator cuff interval fibrosis [46]. The increased presence of nerve tissue biomarker S100 may also be related to the severe pain associated with AC [47].

Current research hypothesizes three main potential pathophysiological mechanisms that lead to AC in a hyperglycemic environment [48]. First, the accumulation of AGEs results in collagen cross-linking, causing capsule fibrosis. Secondly, hyperglycemia promotes the chronic release of inflammatory mediators such as tumor necrosis factor-alpha (TNF-α) and interleukin-6 (IL-6), creating a pro-inflammatory environment that leads to the accumulation of collagen and extracellular matrix, ultimately resulting in fibrosis. Additionally, increased adipocytes in diabetic patients secrete interleukin-13 (IL-13), which is believed to contribute to synovial and connective tissue fibrosis. Free fatty acids secreted by adipocytes can also inhibit their clearance by macrophages and promote collagen deposition in the glenohumeral capsule [49].

Among the various hypothesized pathophysiological mechanisms, AGEs are widely recognized as the most significant influencing factor. The accumulation of AGEs accelerates the cross-linking of collagen and increases resistance to proteolysis, thereby promoting fibroblast proliferation [44]. Since glycation, the process of binding monosaccharides with proteins, is increased by high blood sugar levels, AGEs are considered a key factor in DM-induced AC [50]. In addition to high blood sugar levels, AGEs also increase proinflammatory cytokines and growth factors [51], thereby enhancing the risk of AC.

Interestingly, a rat experiment conducted by Thomas indicated that chronic inflammation caused by high blood sugar levels may also induce collagen changes. Additionally, compensatory hyperinsulinemia [52] and low-grade inflammation [53] associated with DM are related to the accumulation of AGEs [41]. This illustrates the complex and inseparable relationship between high blood glucose levels and AC.

Although metabolic factors such as aging, obesity, and a lack of exercise may also lead to similar AGEs pathological processes, the accumulation of AGEs due to long-term hyperglycemia could well explain the slow progression of AC. The complex pathological mechanisms between these two diseases highlight the need for systemic treatment for AC patients with DM, and it also deserves more investigations.

### 4.2. The Impact of Hyperglycemia on the Musculoskeletal System

Compared to the unclear pathological mechanisms between DM and AC, the effects of high blood sugar levels on the musculoskeletal system, tendons, and ligaments have more supporting evidence and research. Clinically, we already understand that DM increases the destruction and degeneration of the musculoskeletal system, such as with long head biceps tenosynovitis and subacromial-subdeltoid bursitis [54]. Additionally, patients with DM show a significant reduction in shoulder range of motion (SROM) compared to others of the same age.

Recently, Struyf reviewed the research on the impact of DM on the musculoskeletal system. He found that diabetes leads to an abnormal accumulation of collagen layers in the muscles, which increases the thickness of the supraspinatus tendon and the long head of the biceps tendon [48]. This conclusion has been supported by some animal studies, where a chronic hyperglycemic environment was found to increase the diameter and stiffness of the Achilles tendon in mice. On the other hand, an excessive accumulation of extracellular matrix (ECM) proteins is often observed in the tissues of DM patients [55]. Interestingly, in obese diabetic mice, increased collagen deposition in the myocardial stromal cells and the proliferation of collagen-producing cells have also been observed [48,55].

Although these studies do not perfectly explain the current diagnostic features of AC on imaging, such as the thickening of the glenohumeral joint capsule in the axillary pouch (AP) and the thickening of the coracohumeral ligament (CHL), they do reaffirm the impact of chronic hyperglycemia on the muscles, tendons and ligaments. Since the pathological mechanisms of AC remain unclear, we might be able to use the aforementioned clinical and animal studies as a reference and direction for future research. This could ultimately help to unravel the close and mysterious relationship between DM and AC.

### 4.3. The Prevalence of AC and the Regional Difference in DM

In our narrative study, we conducted a more detailed investigation into the impact of general DM, type 1 DM, and blood sugar levels on the prevalence of adhesive capsulitis. According to WHO data, 8.5% of adults in the general population suffer from DM, and 2–5% of adults suffer from AC. Therefore, discussing the association of the prevalence between these two conditions is highly important and practical.

Since AC was first introduced by Codman in 1934, numerous studies have accumulated regarding the disease, and the prevalence of AC in DM patients has been a hot topic at the same time. Despite a remarkable meta-analysis published in 2016, which estimated the AC prevalence to be 13.4% in DM patients [12], studies with differing opinions have emerged since 2015. Some of these studies involved large sample sizes, numbering in the millions. Interestingly, in recent years, studies supporting a strong positive correlation between DM and AC have been predominantly concentrated in Asia. For example, regarding the prevalence of AC among DM patients, a study of the Pakistani population by Inayat reported a rate of 41.3% [29]. Furthermore, when examining the prevalence of DM in AC patients, studies supporting a strong positive correlation are also concentrated in Asia. For instance, research on the Iraqi population by Alhashimi showed a prevalence of 90.3% [23]. Other studies by Pandey and Rai on the Indian population reported rates of 37.5% and 27.4%, respectively [24,30]. This leads us to reasonably speculate that there may be regional statistical differences in the prevalence of AC in DM patients.

A pooled analysis indicates that, due to economic considerations, low- and middle-income countries frequently use fasting plasma glucose (FPG) for diabetes screening. However, this approach may lead to a delayed diagnosis, resulting in organ damage from prolonged exposure to hyperglycemia [56]. This could help to explain why diabetes patients in certain regions are at a higher risk of AC disease and diabetes-related complications. This perspective is also supported by another study by Pandey, who noted that the prevalence of prediabetes in AC cases ranged from 8% to 48% in previous studies [30]. Such significant statistical differences could also stem from the regional variations in DM. Although not all studies are consistent with the regional differences that we observed, the disparities shown in recent studies have become more pronounced compared to those from studies conducted before 2015.

### 4.4. The Correlation between AC and the Chronic Course of DM

As we dig deeper into these highly prevalent diseases, it becomes more valuable to not only identify AC from DM cases, but also to screen for DM and related complications in AC cases. In the past, AC was considered a potential early warning sign of prediabetes [12]. Previous studies have shown that the prevalence of DM in AC patients can be as high as 20–40%. Recently, some studies have continued to support the idea of screening for prediabetes in AC patients, emphasizing that HbA1c is a more effective marker for prediabetes screening compared to random blood sugar (RBS) [24,30,56].

However, other studies suggest that screening for diabetes among AC patients is meaningless [19,21]. The course of DM is often long, so by the time AC is diagnosed, DM may have already been identified. Additionally, two other studies indicate that AC patients are often diagnosed during the chronic stage of diabetes, highlighting the significant impact of long-term hyperglycemia on the development of AC [23,31]. Interestingly, this conclusion aligns with previous biological research suggesting that chronic hyperglycemia can alter the musculoskeletal system through the accumulation of inflammatory substances and AGEs [28].

Previous research by Balci found a significant association between AC and diabetic retinopathy when examining the relationship between AC and chronic DM complications [57]. Another study by Yian even demonstrated a significant correlation between AC and chronic diabetic comorbidities such as kidney disease, peripheral neuropathy, and retinopathy [37]. Therefore, while the use of AC to screen for prediabetes remains a topic of debate, when diagnosing an AC patient, greater attention should be given to the possibility of chronic diabetes and other chronic organ comorbidities.

## 5. Conclusions

In summary, clinicians still need to be aware of the potential risk of DM when treating AC patients, and vice versa. Given the increasing prevalence of DM, special caution is warranted when managing patients with poor blood sugar control, T1DM, and chronic DM, especially for shoulder conditions. Our study compiled research spanning over 30 years and synthesized the data on DM-related AC across different time periods. This provides clinicians with risk and prognostic indicators to consider when dealing with DM patients of varying types and stages.

## Figures and Tables

**Table 3:** Summary of studies about the prevalence of AC and blood sugar level.

Study	DM Types	Methods	Results
Doria et al. (2017) [34]	T1DM	A cross-sectional study on 943 patients with T1DM.	(1) Prevalence of AC in T1DM: 35.1%. (2) Association with Duration: Longer duration of T1DM associated with higher AC prevalence. (3) HbA1c Levels: No significant difference between patients with and without AC. (4) Control of DM: AC more frequent in poorly controlled DM (HbA1c > 8%), but the association was not significant.
Inayat etal. (2017) [29]	Not distinguished	A cross-sectional study on 80 patients.	(1) Prevalence of AC: 41.3% in DM patients. (2) Uncontrolled DM: 1.5 times higher AC rate. (3) Insulin Use: 1.2 times higher AC rate.
Chan et al. (2017) [39]	Not distinguished	A retrospective study on 24,417 patients with DM.	(1) HbA1c Increase: Each unit increase in HbA1c is associated with a 2.77% increase in AC prevalence. (2) AC Patients with DM: 32.2%.
Park et al. (2020) [40]	Not distinguished	A retrospective study on 151 patients with and 453 without AC.	Fasting Glucose Levels and AC Association (1) A total of <85 mg/dL Quartile: Significantly negatively associated with AC. (2) A total of 90–94 mg/dL Quartile or Higher: Significantly positively associated with AC.
Eckert et al. (2021) [18]	T1DM, T2DM	A retrospective study on 44,613 subjects with T1DM and 401,816 with T2DM from DPV database.	(1) Prevalence of AC: Higher in T1DM (0.22%) compared to T2DM (0.06%). (2) Blood Sugar Level: Higher blood sugar levels in T1DM were associated with increased AC prevalence.
Green et al. (2024) [36]	Not distinguished	A Mendelian Randomization study on 379,708 patients in UK Biobank’s unrelated European cohort.	(1) Long-term hyperglycemia has a causal role in upper limb pathologies. (2) Genetical Prediction: A 10 mmol/mol increase in HbA1c is associated with increased risk of AC: OR = 1.50.

## References

[1] Itoi E., Arce G., Bain G.I., Diercks R.L., Guttmann D., Imhoff A.B., Mazzocca A.D., Sugaya H., Yoo M.Y.-S. (2016). Shoulder Stiffness: Current Concepts and Concerns. Arthroscopy.

[2] Pandey V., Madi S. (2021). Clinical Guidelines in the Management of Frozen Shoulder: An Update!. Indian J. Orthop..

[3] Dias R., Cutts S., Massoud S. (2005). Frozen shoulder. Br. Med. J..

[4] Stella S.M., Gualtierotti R., Ciampi B., Trentanni C., Sconfienza L.M., Del Chiaro A., Pacini P., Miccoli M., Galletti S. (2022). Ultrasound Features of Adhesive Capsulitis. Rheumatol. Ther..

[5] Pimenta M., Vassalou E.E., Cardoso-Marinho B., Klontzas M.E., Dimitri-Pinheiro S., Karantanas A.H. (2023). The role of MRI and Ultrasonography in Diagnosis and Treatment of Glenohumeral Joint Adhesive Capsulitis. Mediterr. J. Rheumatol..

[6] Cohen C., Tortato S., Silva O.B.S., Leal M.F., Ejnisman B., Faloppa F. (2020). Association between Frozen Shoulder and Thyroid Diseases: Strengthening the Evidences. Rev. Bras. Ortop..

[7] Hsu K.C., Sun C.H., Wu Y.Y., Chen L.C., Wu Y.T., Chien W.C. (2018). Increased risk of adhesive capsulitis among patients with gout: A nationwide population-based matched-cohort study. Int. J. Rheum. Dis..

[8] Tzeng C.Y., Chiang H.Y., Huang C.C., Lin W.S., Hsiao T.H., Lin C.H. (2019). The impact of pre-existing shoulder diseases and traumatic injuries of the shoulder on adhesive capsulitis in adult population: A population-based nested case-control study. Medicine.

[9] Wang J.Y., Liaw C.K., Huang C.C., Liou T.H., Lin H.W., Huang S.W. (2021). Hyperlipidemia Is a Risk Factor of Adhesive Capsulitis: Real-World Evidence Using the Taiwanese National Health Insurance Research Database. Orthop. J. Sports Med..

[10] Huang S.W., Wang J.Y., Lin C.L., Huang C.C., Liou T.H., Lin H.W. (2020). Patients with Axial Spondyloarthritis Are at Risk of Developing Adhesive Capsulitis: Real-World Evidence Database Study in Taiwan. J. Clin. Med..

[11] Aïm F., Chevallier R., Marion B., Klouche S., Bastard C., Bauer T. (2022). Psychological risk factors for the occurrence of frozen shoulder after rotator cuff repair. Orthop. Traumatol. Surg. Res..

[12] Zreik N.H., Malik R.A., Charalambous C.P. (2016). Adhesive capsulitis of the shoulder and diabetes: A meta-analysis of prevalence. Muscles Ligaments Tendons J..

[13] Whelton C., Peach C.A. (2018). Review of diabetic frozen shoulder. Eur. J. Orthop. Surg. Traumatol..

[14] Dyer B.P., Burton C., Rathod-Mistry T., Blagojevic-Bucknall M., van der Windt D.A. (2021). Diabetes as a Prognostic Factor in Frozen Shoulder: A Systematic Review. Arch. Rehabil. Res. Clin. Transl..

[15] Cho C.H., Jin H.J., Kim D.H. (2020). Comparison of Clinical Outcomes between Idiopathic Frozen Shoulder and Diabetic Frozen Shoulder After a Single Ultrasound-Guided Intra-Articular Corticosteroid Injection. Diagnostics.

[16] Tawfeek W., Addosooki A., Elsayed M. (2022). Arthroscopic rotator interval release for frozen shoulder, comparative study between diabetic and non-diabetic patients. Sicot. J..

[17] Yanlei G.L., Keong M.W., Tijauw Tjoen D.L. (2019). Do diabetic patients have different outcomes after arthroscopic capsular release for frozen shoulder?. J. Orthop..

[18] Eckert A.J., Plaumann M., Pehlke S., Beck C., Mühldorfer S., Weickert U., Laimer M., Pfeifer M., Stechemesser L., Holl R. (2022). Idiopathic Frozen Shoulder in Individuals with Diabetes: Association with Metabolic Control, Obesity, Antidiabetic Treatment and Demographic Characteristics in Adults with Type 1 or 2 Diabetes from the DPV Registry. Exp. Clin. Endocrinol. Diabetes.

[19] Safran O., El-Haj M., Leibowitz G., Beyth S., Furman Z., Milgrom C., Kandel L. (2017). Should Patients With Frozen Shoulder Be Screened for Diabetes Mellitus?. Orthop. J. Sports Med..

[20] Kim J.H., Kim B.S., Han K.D., Kwon H.S. (2023). The Risk of Shoulder Adhesive Capsulitis in Individuals with Prediabetes and Type 2 Diabetes Mellitus: A Longitudinal Nationwide Population-Based Study. Diabetes Metab. J..

[21] Gundtoft P.H., Kristensen A.K., Attrup M., Vobbe J.W., Luxhøi T., Rix F.G., Hölmich P., Sørensen L. (2018). Prevalence and Impact of Diabetes Mellitus on the Frozen Shoulder. South. Med. J..

[22] Jacob L., Gyasi R.M., Koyanagi A., Haro J.M., Smith L., Kostev K. (2023). Prevalence of and Risk Factors for Adhesive Capsulitis of the Shoulder in Older Adults from Germany. J. Clin. Med..

[23] Alhashimi R.A.H. (2018). Analytical Observational Study of Frozen Shoulder among Patients with Diabetes Mellitus. Joints.

[24] Rai S.K., Kashid M., Chakrabarty B., Upreti V., Shaki O. (2019). Is it necessary to screen patient with adhesive capsulitis of shoulder for diabetes mellitus?. J. Family Med. Prim. Care.

[25] Buchbinder R., Green S. (2004). Effect of arthrographic shoulder joint distension with saline and corticosteroid for adhesive capsulitis. Br. J. Sports Med..

[26] Shah N., Lewis M. (2007). Shoulder adhesive capsulitis: Systematic review of randomised trials using multiple corticosteroid injections. Br. J. Gen. Pract..

[27] Boyle-Walker K.L., Gabard D.L., Bietsch E., Masek-VanArsdale D.M., Robinson B.L. (1997). A profile of patients with adhesive capsulitis. J. Hand Ther..

[28] Dyer B.P., Rathod-Mistry T., Burton C., van der Windt D., Bucknall M. (2023). Diabetes as a risk factor for the onset of frozen shoulder: A systematic review and meta-analysis. BMJ Open.

[29] Inayat F., Ali N.S., Shahid H., Younus F. (2017). Prevalence and Determinants of Frozen Shoulder in Patients with Diabetes: A Single Center Experience from Pakistan. Cureus.

[30] Pandey V., Aier S., Agarwal S., Sandhu A.S., Murali S.D. (2024). Prevalence of prediabetes in patients with idiopathic frozen shoulder: A prospective study. JSES Int..

[31] Juel N.G., Brox J.I., Brunborg C., Holte K.B., Berg T.J. (2017). Very High Prevalence of Frozen Shoulder in Patients With Type 1 Diabetes of ≥45 Years’ Duration: The Dialong Shoulder Study. Arch. Phys. Med. Rehabil..

[32] Arkkila P.E., Kantola I.M., Viikari J.S., Rönnemaa T. (1996). Shoulder capsulitis in type I and II diabetic patients: Association with diabetic complications and related diseases. Ann. Rheum. Dis..

[33] Bunker T.D. (1997). Frozen shoulder: Unravelling the enigma. Ann. R. Coll. Surg. Engl..

[34] Doria C., Mosele G.R., Badessi F., Puddu L., Caggiari G. (2017). Shoulder Adhesive Capsulitis in Type 1 Diabetes Mellitus: A Cross-Sectional Study on 943 Cases in Sardinian People. Joints.

[35] Yang W., Yang Y., Han B. (2023). Socioeconomic status, obesity, individual behaviors, diabetes, and risk for frozen shoulder: A Mendelian randomization study. Medicine.

[36] Green H.D., Jones A., Evans J.P., Wood A.R., Beaumont R.N., Tyrrell J., Frayling T.M., Smith C., Weedon M.N. (2021). A genome-wide association study identifies 5 loci associated with frozen shoulder and implicates diabetes as a causal risk factor. PLoS Genet..

[37] Yian E.H., Contreras R., Sodl J.F. (2012). Effects of glycemic control on prevalence of diabetic frozen shoulder. J. Bone Joint Surg. Am..

[38] Lin B.S., Chien W.C., Lu C.H., Chung C.H., Tsao C.H., Weng T.H., Lin C.Y. (2024). Exploring the link between metformin use and adhesive capsulitis of the shoulder: A retrospective cohort study in Taiwan. Naunyn Schmiedebergs Arch. Pharmacol..

[39] Chan J.H., Ho B.S., Alvi H.M., Saltzman M.D., Marra G. (2017). The relationship between the incidence of adhesive capsulitis and hemoglobin A(1c). J. Shoulder Elbow Surg..

[40] Park H.B., Gwark J.Y., Kam M., Jung J. (2020). Association between fasting glucose levels and adhesive capsulitis in a normoglycemic population: A case-control study. J. Shoulder Elbow Surg..

[41] de la Serna D., Navarro-Ledesma S., Alayón F., López E., Pruimboom L. (2021). A Comprehensive View of Frozen Shoulder: A Mystery Syndrome. Front. Med..

[42] Kabbabe B., Ramkumar S., Richardson M. (2010). Cytogenetic analysis of the pathology of frozen shoulder. Int. J. Shoulder Surg..

[43] Ryu J.D., Kirpalani P.A., Kim J.M., Nam K.H., Han C.W., Han S.H. (2006). Expression of vascular endothelial growth factor and angiogenesis in the diabetic frozen shoulder. J. Shoulder Elbow Surg..

[44] Hwang K.R., Murrell G.A., Millar N.L., Bonar F., Lam P., Walton J.R. (2016). Advanced glycation end products in idiopathic frozen shoulders. J. Shoulder Elbow Surg..

[45] Holte K.B., Juel N.G., Brox J.I., Hanssen K.F., Fosmark D.S., Sell D.R., Monnier V.M., Berg T.J. (2017). Hand, shoulder and back stiffness in long-term type 1 diabetes; cross-sectional association with skin collagen advanced glycation end-products. The Dialong study. J. Diabetes Complicat..

[46] Cho C.H., Song K.S., Kim B.S., Kim D.H., Lho Y.M. (2018). Biological Aspect of Pathophysiology for Frozen Shoulder. Biomed. Res. Int..

[47] Hand G.C., Athanasou N.A., Matthews T., Carr A.J. (2007). The pathology of frozen shoulder. J. Bone Joint Surg. Br..

[48] Struyf F., Mertens M.G., Navarro-Ledesma S. (2022). Causes of Shoulder Dysfunction in Diabetic Patients: A Review of Literature. Int. J. Environ. Res. Public. Health.

[49] Dimitri-Pinheiro S., Pimenta M., Cardoso-Marinho B., Torrão H., Soares R., Karantanas A. (2021). Diabetes: A silent player in musculoskeletal interventional radiology response. Porto Biomed. J..

[50] Lebiedz-Odrobina D., Kay J. (2010). Rheumatic manifestations of diabetes mellitus. Rheum. Dis. Clin. N. Am..

[51] Schmidt A.M., Yan S.D., Wautier J.L., Stern D. (1999). Activation of receptor for advanced glycation end products: A mechanism for chronic vascular dysfunction in diabetic vasculopathy and atherosclerosis. Circ. Res..

[52] Hsu C.L., Sheu W.H. (2016). Diabetes and shoulder disorders. J. Diabetes Investig..

[53] Pietrzak M. (2016). Adhesive capsulitis: An age related symptom of metabolic syndrome and chronic low-grade inflammation?. Med. Hypotheses.

[54] Abate M., Schiavone C., Salini V. (2010). Sonographic evaluation of the shoulder in asymptomatic elderly subjects with diabetes. BMC Musculoskelet. Disord..

[55] Tuleta I., Frangogiannis N.G. (2021). Diabetic fibrosis. Biochim. Biophys. Acta Mol. Basis Dis..

[56] (2023). Global variation in diabetes diagnosis and prevalence based on fasting glucose and hemoglobin A1c. Nat. Med..

[57] Balci N., Balci M.K., Tüzüner S. (1999). Shoulder adhesive capsulitis and shoulder range of motion in type II diabetes mellitus: Association with diabetic complications. J. Diabetes Complicat..

